# Update on the Genetics of Osteogenesis Imperfecta

**DOI:** 10.1007/s00223-024-01266-5

**Published:** 2024-08-11

**Authors:** Milena Jovanovic, Joan C. Marini

**Affiliations:** 1 Section on Heritable Disorders of Bone and Extracellular Matrix, Eunice Kennedy Shriver National Institute of Child Health and Human Development, National Institutes of Health, Bethesda, MD USA; 2 Present Address: Section on Adolescent Bone and Body Composition, Eunice Kennedy Shriver National Institute of Child Health and Human Development, National Institutes of Health, Bethesda, MD USA

**Keywords:** Osteogenesis imperfecta, Bone mineralization, Osteoblast differentiation, Mitochondria, IFITM5/BRIL, PDEF, RIP/MBTPS2, MAPK/ERK

## Abstract

Osteogenesis imperfecta (OI) is a heterogeneous heritable skeletal dysplasia characterized by bone fragility and deformity, growth deficiency, and other secondary connective tissue defects. OI is now understood as a collagen-related disorder caused by defects of genes whose protein products interact with collagen for folding, post-translational modification, processing and trafficking, affecting bone mineralization and osteoblast differentiation. This review provides the latest updates on genetics of OI, including new developments in both dominant and rare OI forms, as well as the signaling pathways involved in OI pathophysiology. There is a special emphasis on discoveries of recessive mutations in *TENT5A*, *MESD*, *KDELR2* and *CCDC134* whose causality of OI types XIX, XX, XXI and XXI, respectively, is now established and expends the complexity of mechanisms underlying OI to overlap LRP5/6 and MAPK/ERK pathways. We also review in detail new discoveries connecting the known OI types to each other, which may underlie an eventual understanding of a final common pathway in OI cellular and bone biology.

## Introduction

Osteogenesis imperfecta (OI) or “brittle bone disease” is a genetically and phenotypically heterogeneous heritable skeletal disorder with an incidence of 1:15,000–20,000 live births. OI is primarily characterized by bone fragility and deformity, growth deficiency and highly variable secondary connective tissue findings, including blue sclerae, dentinogenesis imperfecta (DI), cardiovascular and respiratory defects, hearing loss, joint hypermobility. OI inheritance patterns include autosomal dominant and recessive, as well as an X-linked recessive form. The majority of individuals with OI (80–85% in Western countries; about 60% in countries with a high incidence of consanguinity) are caused by structural or quantitative defects in collagen type I genes, *COL1A1* and *COL1A2*, encoding the α1(I) and α2(I) chains of type I collagen, respectively. However, OI is now understood to be a collagen-related disorder, with discovery of OI types caused by defects in genes whose protein products interact with collagen for folding, post-translational modification, processing and trafficking, as well as gene defects that affect primarily bone mineralization and osteoblast differentiation [1, 2].

The Sillence Classification of OI, proposed in 1979, was the first useful nosology. It divided OI into four “types” based on phenotypic (with a strong emphasis on scleral hue) and radiographic characteristics, as well as on the severity of bone fragility. Type I was the mildest group, generally with fractures in childhood but requiring clinical examination and radiographs to distinguish from individuals without OI. Type II was clinically severe, perinatal lethal individuals, who also had undertubulation of long bones on radiographs, generally with prenatal fractures of long bone and ribs and dark blue to blue-grey sclerae. Type III OI represented the severe non-lethal form, called “progressive deforming”. They fracture easily, require surgery and assistive devices for mobility, have striking short stature, barrel chest and scoliosis, and, frequently, relative macrocephaly. Type IV is the moderately severe form. These individuals have childhood fractures that lessen in frequency in adolescence. They have notable short stature and may develop scoliosis but can generally be independent ambulator with placement of intramedullary rods. The Sillence Classification was published before any of the genetic causes of OI were determined and proposed that there would be both dominant and recessive causes, with a greater severity in recessive cases.

In the subsequent decades, structural or quantitative defects in type I collagen were identified in the majority of individuals with OI. It emerged that most individuals with mild type I OI had dominant mutations in *COL1A1* causing an effectively null allele and leading to the production of a reduced amount of structurally normal collagen in bone matrix. Mutations in *COL1A1* and *COL1A2*, predominantly substitutions for glycine residues that uniformly occur at every third position of the collagen helical region or splicing defects, cause the full phenotypic range of types II–III–IV OI. Genotype–phenotype correlation studies have shown some alignment of specific collagen mutations with OI type. However, there is considerable variability of clinical outcome of mutations at the same site and molecular overlap of mutations with very different outcome. For these reasons, the Sillence types are still first a clinical classification.

After 2006, various advances led to the identification of multiple other genes causing OI and leading to a Genetic Classification. To date, the Genetic Classification describes 22 OI types. Type I–IV OI are reserved for those individuals with dominant defects in type I collagen genes. Type I OI is reserved for the subset of individuals whose collagen genes lead to a quantitative defect of type I collagen. Types II–III–IV are based on the Sillence types, that is, while these individuals all have collagen structural defects, the OI type assignment is based on the same phenotypic features as in the original Sillence nosology.

Types V and VI OI were defined histologically before their gene defect was known. They are characterized by altered bone mineralization, caused by a recurrent dominant mutation in *IFITM5* in type V, recessive null mutations in *SERPINF1* in VI, and *IFITM5*/BRIL p.S40L in atypical VI (aVI), respectively. Recessive defects in *CRTAP*, *P3H1* and *PPIB* underlie absence of the procollagen prolyl 3-hydroxylation complex components, causing OI types VII, VIII and IX respectively. Recessive mutations in *SERPINH1*, *FKBP10* and *BMP1* affecting collagen processing and cross-linking, cause OI types X, XI and XIII, respectively. Impaired osteoblasts differentiation is a common features of OI types XII, XIV, XV, XVI, XVII, XVIII, caused by recessive mutations in *SP7*, *TMEM38B*, *WNT1*, *CREB3L1*, *SPARC* and *MBTPS2*. *WNT1* also causes less severe dominantly inherited bone fragility. The recent discoveries about the bone mechanisms of newly established OI types XIX, XX, XXI and XXII have fully validated recessive mutations in *TENT5A*, *MESD*, *KDELR2* and *CCDC134,* respectively, as OI-causative within the umbrella of type I collagen-related gene defects.

**Fig. 1:**
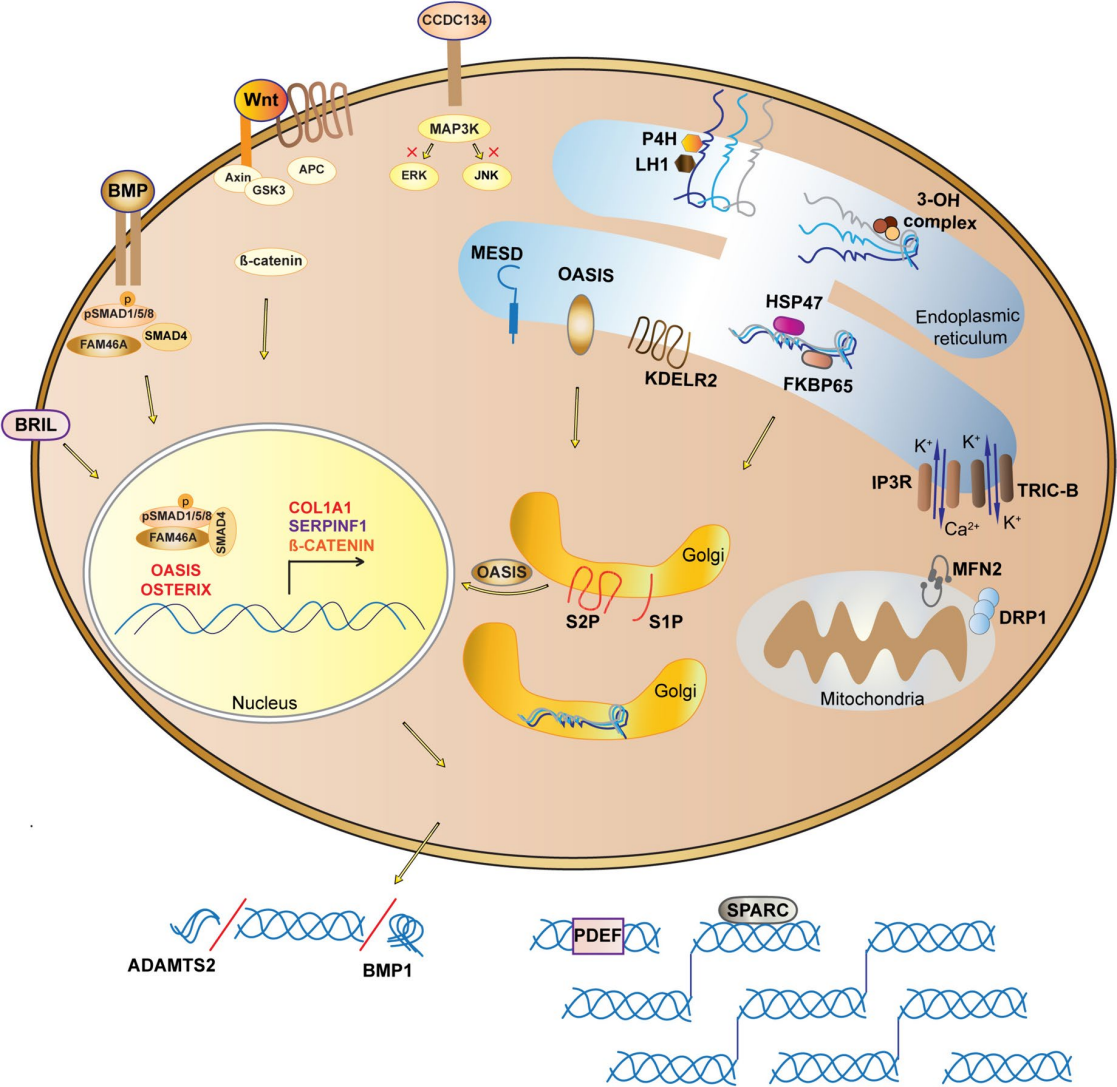
Mechanisms and signaling pathways of OI. The two proα1(I) and one proα2(I) chains of the procollagen heterotrimer undergo post-translational modifications of proline and lysine residues by P4H and LH1, respectively, in the ER. During procollagen folding, the ER-residing prolyl 3-OH complex, consisting of P3H1, CRTAP, and PPIB, hydroxylates the P986 residue important for alignment of procollagen chains. HSP47, an ER chaperone, binds to procollagen triple helix to facilitate collagen folding and secretion. FKBP10, another ER chaperone, cooperate with HSP47 in procollagen trafficking from the ER to the Golgi. Another protein that functions closely with HSP47 is KDEL receptor 2, which has a role in intracellular recycling of ER-resident proteins through retrograde transport. Once the procollagen triple helix is secreted into extracellular space, N- and C-propeptides of procollagen are cleaved by ADAMTS-2 and BMP1 enzymes, respectively. Type I collagen is released and incorporated into extracellular matrix. An important regulator of collagen assembly is SPARC, which binds collagen as a matricellular glycoprotein. At the level of transcriptional regulation, Osterix/Sp7, a zinc finger transcriptional factor, interacts with RUNX2, which induces the expression of collagen genes. Intracellular calcium is important for collagen post-translational modification and metabolism. TRIC-B together with IP3R regulate calcium flux from the ER to cytoplasm. In addition, TRIC-B absence impacts the fusion and fission of mitochondria, affecting MFN2 and DRP1 regulators at the ER-mitochondria contact sites. Transmembrane transcriptional factor OASIS is transported from the ER to the Golgi membrane for RIP signaling. OASIS is cleaved by S1P and S2P in the Golgi and the released N-terminal end of OASIS translocates into the nucleus for transcription of genes important for extracellular matrix, such as collagens. Also affecting collagen expression and bone mineralization in osteoblasts is FAM46A, encoded by *TENTA*, which is involved in the polyadenylation of collagen transcripts and other proteins involved in bone mineralization. BRIL is an important regulator of bone mineralization and impacts *SERPINF1* transcription in the nucleus. *SERPINF1* encodes the PDEF protein, which binds collagen to activate its own anti-angiogenic function. Wnt signaling, important for bone development, is activated by binding of WNT1 ligand to the Frizzled and LRP5/6 receptors, which inhibits the degradation complex, consisting of Axin, APC, and GSK3, in order to stabilize β-catenin. β-catenin translocates to the nucleus to activate transcription of Wnt target genes. MESD, a chaperone residing in the ER, has a role in Wnt signaling by translocating LRP5/6 receptors, and is also a direct chaperone of pro-α1(I) in osteoblasts. BMP signaling is another important pathway for bone metabolism. BMP binds to BMP receptors and induces phosphorylation of SMAD 1/5/8. Together SMAD 1/5/8, FAM46A, and SMAD4 form a complex that further translocates to the nucleus to induce transcription of BMP target genes. Recently proposed OI-causing gene *CCDC134* encodes a secretory protein CCDC134 that inhibits ERK and JNK phosphorylation of MAPK pathway, and impacts osteoblast expression of collagen and mineralization

The Genetic Classification is not simply a list of genes in which defects cause osteogenesis imperfecta. In fact, largely driven by the process of rare disease gene identification, the genes were identified in logical “clusters” by mechanism. For example, when one component of the procollagen prolyl 3-hydroxylation complex was identified in recessive OI, investigators naturally looked quickly to the other two components. Or when a skeletal phenotype in the murine model for *CREB3L1*/OASIS led to children with rare recessive defects in OASIS, it becomes logical that defects in its processing protease, S2P, encoded by *MBTPS2*, could also cause OI. Sometimes serendipity intervenes and the related gene is not the next one identified, but their proximity in the gene list leads to their presence in the same “mechanism group”, thus serving both as an aide to memory and a guide to further research. Some (predominantly clinical) investigators wish to fold the new genes into the phenotypic classification based on the Sillence nosology. This is said to have the advantage of being simpler for clinicians and families, and to result in more uniform treatment based on phenotype. However, this simplicity masks some obvious and some latent opportunities for inaccuracy in a purely phenotypic classification. The OI patients in each Sillence phenotypic type will have multiple different genetic etiologies to their bone dysplasia, reflecting different mechanisms and likely/possibly responding differently to the same pharmacological or non-pharmacological treatment. Parents of children with the same rare condition are connected to each other via social media and OI-clinics; they are astute in figuring out which patient is different and are reasonably interested in understanding the differences and their implications. A second factor is the fluidity and variability of phenotype. Individuals, even in the same family, with mutations in the same gene may have sufficient difference in severity to be assigned to a different phenotypic type. Or phenotype my worsen over the decades of a life, changing the assigned type. These factors would confuse patients, families and primary doctors, and potentially muddling treatment protocols. The best of both genetic and phenotypic systems can be obtained by having the Genetic Classification as the primary type, with a secondary designation for the clinical severity of the phenotype which could change with time and circumstance. The genetic classification of OI types is listed in Table 1, with mechanisms and signaling pathways presented in Fig. 1.

**Table 1:** The genetic classification of OI types

OI type	Inheritance	Gene	Protein	Severity	OI classical and distinguishing features
Collagen structure					
I	AD	*COL1A1*	Collagen ɑ1	Mild	Quantitative defect due to loss of function of one of COL1A1 alleles
II–IV	AD	*COL1A1*, *COL1A2*	Collagen ɑ1 or ɑ2	Lethal, severe to moderate	Structural defects in collagen helix or C-propeptides
Bone mineralization					
V	AD	*IFITM5*	BRIL (BRIL5' MALEP)	Moderate	Hyperplastic callus formation, forearm interosseous membrane ossification, radial head dislocation, forearm dense metaphyseal bands, mesh like pattern of lamellar bone
Atypical VI	AD	*IFITM5*	BRIL (BRILp.S42L)	Severe progressive	Accumulated osteoid, fish scale pattern of lamellar bone, increased ALPL serum levels in childhood
VI	AR	*SERPINF1*	PEDF	Severe progressive	PEDF deficiency, accumulated osteoid, fish scale pattern of lamellar bone, increased ALP serum levels in childhood
Collagen modification					
VII	AR	*CRTAP*	CRTAP	Lethal to severe	Absent procollagen prolyl 3-hydroxylation, rhizomelia
VIII	AR	*LERPE1*	P3H1	Lethal to severe	Absent procollagen prolyl 3-hydroxylation, rhizomelia
IX	AR	*PPIB*	CyPB	Lethal to moderate	Absent procollagen prolyl 3-hydroxylation, no rhizomelia, dental anomalies
Collagen folding and crosslinking					
X	AR	*SERPINH1*	HSP47	Lethal to severe	Respiratory defects, blue sclerae, Wormian bones
XI	AR	*FKBP10*	FKBP65	Moderate	Cause of Bruck or Kuskokwim syndromes
XIII	AR	*BMP1*	BMP1	Mild to severe	Deficiency of C-propeptidase
XXI*	AR	*KDELR2*	KDEL receptor 2	Severe progressive	Multiple fractures, chest deformity, short stature
Osteoblast function and differentiation					
XII	AR	*SP7*	OSTERIX	Moderate	Delayed tooth eruption, craniofacial anomalies
XIV	AR	*TMEM38B*	TRIC-B	Asymptomatic to severe	Altered mitochondrial function and cell adhesion
XV	AD/AR	*WNT1*	WNT1	Moderate	Cause of OI or early-onset osteoporosis, variable neurological defects
XVI	AR	*CREB3L1*	OASIS	Lethal to mild	Defect in RIP pathway
XVII	AR	*SPARC*	SPARC	Severe progressive	Muscular hypotonia
XVIII	XR	*MBTPS2*	S2P	Moderate	Defect in RIP pathway
Newly classified OI types					
XIX	AR	*TENT5A*	FAM46A	Lethal to severe	Defect in BMP/TGFβ signaling pathway
XX	AR	*MESD*	MESD	Lethal to severe	LRP5/6 related disorder
XXI*	AR	*KDELR2*	KDEL receptor 2	Severe progressive	Multiple fractures, chest deformity, short stature
XXII	AR	*CCDC134*	CCDC134	Severe	Dysregulation of the MAPK/ERK pathway

*AD* autosomal dominant, *AR* autosomal recessive, *XR* X-linked recessive

*Type XXI OI is listed twice in this table, once for its functional role and once with newly identified types. Types XVIII and XIX OI are listed as types XIX and XVIII, respectively, in OMIM

The clinical presentation of OI ranges from mild to perinatal lethal. Type I OI is the mildest form of OI, which is caused by quantitative defects in collagen and presents clinically with blue sclerae, variable hearing loss, and bone fractures during childhood which decrease in frequency after puberty. The main features of OI include long bone fractures, growth deficiency and skeletal deformities. However, the OI symptoms go beyond bone fragility, including the secondary defects in other connective tissues such as cardiovascular and pulmonary defects, hearing loss, DI, blue sclerae, Wormian bones, and joint hypermobility.

OI types I-IV due to mutations in the helical, N-propeptide, and C-propeptide regions of *COL1A1* and *COL1A2*, and OI Type XIII due to mutations in *BMP1.*

## OI Types I-IV Due to Mutations in the Helical, N-Propeptide, and C-Propeptide Regions of *COL1A1* and *COL1A2*, and OI Type XIII Due to Mutations in *BMP1*

### Introduction

Type I collagen is the most abundant protein in the body and the main constituent of the extracellular matrix (ECM) of bone and skin. Type I procollagen heterotrimer consists of two pro-α1 (I) and one pro-α2 (I) chains, synthesized in the endoplasmic reticulum (ER), that undergo post-translational modifications of proline and lysine residues carried out by enzymes prolyl-4-hydroxylase 1 (P4H1) and lysyl hydroxylase 1 (LH1). ER chaperones facilitate the folding and assembly of the triple helix, which is subsequently transported to Golgi membranes and then to the ECM. Mature type I collagen is formed by cleavage of the N- and C-terminal propeptides via metalloproteinases ADAMTS-2 (ADAM metallopeptidase with thrombospondin type 1 motif 2) and BMP1 (bone morphogenetic protein 1), respectively. Mature collagen can then self-assemble into heterotypic fibrils with types III and V collagen.

## Structural and Quantitative Mutations

The most common OI mutations are point mutations which cause the substitution of one of the helical glycines, that occur at every third residue in uninterrupted repeating Gly‐Xaa‐Yaa triplets, by residues with a bulky or charged side chain. Because the glycine residues should normally be accommodated in the sterically restricted internal aspect of the helix, the substitutions cause a slower folding of the collagen, exposing the constituent chains for a longer time to post-translational modification enzymes [3].

Splice site mutations are the second most common type of OI collagen mutations. These may cause exon skipping, intron retention or use of cryptic splice sites. Substitutions of non-glycine residues found at X and Y positions of Gly-Xaa-Yaa triplets are distinct from glycine substitutions. Bone fragility is generally mild, and affected individuals have other connective tissue disorders such as OI/Ehlers-Danlos Syndrome (EDS) or Caffey disease. The most common X or Y position substitution is of an arginine residue by a cysteine, leading to formation of intermolecular disulfide bonds within the collagen helix which cause kinking of the helix and a register shift affecting N-propeptide processing [4–6]. A rare mutation consisting of deletion or duplication of one or two Gly-Xaa-Yaa triplets causes severe or lethal OI. These “register shift” mutations induce helical overmodification and disrupt binding sites for non-collagenous proteins along the collagen helix. [7, 8].

Phenotypic variability is one of the major hallmarks of OI and other skeletal dysplasias. It occurs when OI patients with the same mutation, even siblings, have sufficiently different levels of OI clinical severity that they would be given different phenotypic classifications. This phenomenon obscures the therapy of OI patients and further studies are warranted to explore which signaling pathways and modifying factors contribute to this variability.

The Consortium for OI Mutation Genotype–Phenotype analysis [9, 10] showed that substitutions in the α1(I) chain have more severe and lethal phenotype compared to α2(I), clustered in the major ligand binding regions (MLBR) in the collagen triple helix. While the majority of glycine substitutions in the α2(I) chain were not lethal, more lethal splice site mutations occur in this chain [9]. In a recent genotype–phenotype study of a large cohort, 39 of 217 reported glycine substitutions in α1(I) chain were non-lethal, while 58 glycines had at least one lethal case. In α2(I) chain, 63 of 222 reported glycine substitutions were non-lethal and 49 substitutions had a lethal outcome [10]. In another recent population-based study of 166 patients, six pathogenic variants, with four being newly discovered, were identified in the lethal regions of *COL1A1* and *COL1A2* genes in seven patients with non-lethal OI, adding to the prior reported individuals that indicated not all glycine substitutions located in the lethal domain lead to a fatal outcome [11].

Mutations leading to haploinsufficiency in *COL1A1* cause the mildest form of OI, type I. Haploinsufficiency mutations lead to mRNA instability; the mutant allele is thus unable to produce the collagen protein chain, causing quantitative defects of structurally normal collagen. Typical causative mutations introduce a premature stop codon in the coding region of *COL1A1* mRNA by either single nucleotide substitutions, insertion and/or deletion of nucleotides, or secondary to alternative splicing when a mutation is located at a splice sites [12]. Haploinsufficiency mutations in *COL1A2* result in EDS if the mutation is homozygous or compound heterozygous [13, 14].

### OI Murine Models with Collagen Defects

The Brittle mouse model (Brtl^+/−^) is a model for investigating classical OI. It carries a heterozygous α1(I) Gly349Cys substitution in one of *COL1A1* alleles, and presents with symptoms and findings of dominant OI and variability of phenotype. About one-third of newborn mice die on day P1 due to respiratory distress, while living mice have a fairly uniform moderately severe OI phenotype [15]. Affected mice are small and exhibit low bone mass and hypermineralized bone, altered bone biomechanical properties and bone remodeling as well as increased bone fractures [16–18].

Another mouse model for studying a classical OI is the G610C OI knock-in mouse, with a Gly610Cys substitution in the *COL1A2* gene. G610C, also known as the “Amish mouse”, was created based on the collagen mutation identified in 64 individuals in an Old Order Amish community who had OI type I/IV with considerable variability of phenotype [19]. The mice have low bone mineral density (BMD) and body weight, hypermineralized bone and altered bone biomechanical properties [20].

Oim/oim is an OI mouse model with a spontaneous mutation in *Col1a2* gene with a moderate to severe OI phenotype similar to OI type III. Homozygous mice have a guanine deletion at nucleotide 3983 of *Col1a1* gene which alters the last 48 amino acids sequence of proα2 (I) propeptide and causes the accumulation of α1 (I) homotrimer collagen. The oim/oim mice have skeletal deformities and fractures, osteopenia, and thin cortical bone [21]. However, this model is atypical for collagen mutations in that the phenotype has recessive inheritance and α1(I) homotrimers are not a normal component of bone matrix.

Aga2 (abnormal gait 2) mouse model, with a dominant frameshift mutation in *Col1a1* C-propeptide, is broadly applicable, especially for non-skeletal aspects of dominant OI such as cardiovascular and respiratory defects as well as apoptosis studies [22, 23]. Aga2 mice have two phenotypes, while 34% of newborn mice are lethal due to cardiorespiratory defects, the remaining 66% survive with moderately severe OI with bone fractures, decreased body size and bone mass. Aga2 mice show accumulation of proα1(I) intracellularly that leads to activation of ER-stress-specific unfolded protein response (UPR) and apoptosis of osteoblasts [23].

*Col1a1*^Jrt/+^, or the Jrt model, is the first mouse model of combined OI/EDS phenotype which makes it suitable for investigating the OI/EDS pathophysiology [24]. Jrt mice have a dominant mutation in *Col1a1* causing skipping of exon 9 and leading to an 18 amino acid deletion in the N-terminal domain of *Col1a1*. Mice exhibit reduced bone mass and brittle bones susceptible to fractures as OI characteristics, and reduced skin tensile properties and spine curvature as part of EDS phenotype [24].

The Mov13 mouse was the first murine model to confirm that type I collagen mutations caused OI. Mov13 was created in 1983 by the then-prevalent technique of insertion of murine Moloney leukemia virus into the first intron of the *Col1a1* gene, preventing initiation of *Col1a1* [25]. Homozygous mice produce no type I collagen and die at mid-gestation due to blood vessels rupture, while heterozygous mice survive until adulthood and are a model for type I OI. Heterozygous mice produce 50% less collagen resulting in a 50% decrease of tissue collagen content, which induces bone brittleness [26]. Interestingly, the Mov13 skeleton spontaneously adapted during the first 2 months of life to significantly improved bending strength of long bones [27]. Activation of the provirus at about 4 months of age makes this a difficult mouse to manage, and it is no longer in use.

Generation of a mouse model with a classical collagen glycine substitution in a conditional allele that allows tissue specific expression would be very useful for understanding secondary features of OI.

### Defects in Collagen Processing and Propeptides, *BMP1*

Following synthesis of procollagen chains, they align with each another at the C-propeptide and initiate association and folding toward the N-terminal propeptide. To release mature collagen molecules, N- and C-propeptides are processed by metalloproteinases ADAMTS-2 and BMP1/tolloid-like proteinases, respectively.

A single heterozygous mutation in the signal peptide cleavage site of *COL1A1* has been reported in both an infant with lethal type II OI and in a severe terminated fetus [28, 29]. Cells and tissues from the terminated fetus showed delayed collagen secretion and collagen intracellular retention, leading to decreased collagen production. The intracellular collagen appears to be overmodified despite the position of the mutation, while the secreted portion is electrophoretically normal. ER cisternae in cells from the affected fetus are enlarged. The skeletal pathology is attributed to the retained collagen, with putative impaired cleavage and thus impaired release of pre-procollagen from ER-entry sites. This could lead to misfolding and ER stress from both the mutant procollagen chains, a dominant negative effect on entry of normal α1(I) chains, and impaired generalized entry of other proteins to the ER [29].

Mutations in the first approximately 90 residues of the type I collagen α(I) chains interfere with removal of the N-propeptide, although the primary sequence of the N-proteinase cleavage site is intact, and are causative for an OI/EDS phenotype. This phenotype was first described in a group of children presenting with OI with bright blue sclerae, who also had striking hyperextensibility of both large and small joints, reduced collagen fibril diameters and friable tissue [30]. The severity of the OI associated with OI/EDS varied with the specific residue substituted (types III or IV OI) while the EDS symptoms were less striking with further distance from the cleavage site. A subsequent study began with a cohort of patients primarily referred for EDS symptoms, in whom Gly substitutions and exon skipping defects were found in in the amino-end of the *COL1A1* or *COL1A2* helical region [31]. They shared the delayed N-terminal processing, severe joint hyperlaxity, easy bruising of the cohort presenting predominantly with OI, but some also had signs of vascular fragility, and all had mild OI manifestations with blue sclerae, short stature and few fractures. Thus, mutations in the helical region of either the α1(I) or α2(I) chains adjacent to the N-propeptide cleavage site may present as predominantly OI or EDS.

Substitutions in the type I procollagen C-propeptide cleavage site in *COL1A1* or *COL1A2* were first reported with a mild dominant OI phenotype in patients with normal or increased DXA* z*-score and bone mineralization increased higher than in classical OI on BMDD, and impaired C-propeptide processing [32, 33]. Patients have normal growth, light sclerae, straight spine, normal teeth and hearing. Collagen biochemistry was minimally altered with slight backstreaking of α1(I) and α2(I) chains but with normal thermal stability. Histomorphometry yielded normal trabecular bone volume and thick osteoid seams. Tissue fibrils of collagen were thin with a “barbed-wire” appearance [32]. More recently a larger set of patients affected with high bone mass (HBM) OI was reported [33], supporting the predominantly mild phenotype. Although they had fractures of large long bones that persisted into adulthood, fractures were infrequent. Height was in the lower half of the normal curve; again, sclerae were light, teeth and hearing were normal. The mean spinal *z*-score was + 2.9 SD and qBEI confirmed high levels of bone mineralization. Histomorphometry on two patients confirmed hyperostoidosis. Interestingly, in this older cohort, one-third of subjects older than 15 years had scoliosis.

The C-propeptide itself of type I procollagen is critical for recognition and association of chains to form the triple helix. The C-propeptide is processed in the pericellular space and not incorporated into matrix. Surprisingly, mutations located in the C-propeptide were found to cause mild to lethal OI [34, 35]. The severity of OI phenotype is generally correlated with the structure of the triplet C-propeptide, sometimes compared to a flower, with a base or stem, a transition region, and 4 “petals”, as well as multiple interchain bonds [34]. Mutations that affect areas of interaction between chains or calcium-binding sites lead to a severe consequence. Most but not all mutations in outward facing residues lead to a milder type of OI [34, 35]. These mutations lead to both osteoblast intracellular and matrix dysfunction. Immunofluorescence microscopy indicated that procollagen with C-propeptide defects was mislocalized to the ER lumen, in contrast to the ER membrane localization of normal procollagen and procollagen with a classical collagen helical defect [35]. Slower processing of the mutant C-propeptide leads to pC-collagen incorporation into matrix [35]. Finally, there is a quantitative defect that occurs when nonsense-mediated mRNA decay (NMD) reduces the synthesis of mutant procollagen, leading to a milder OI phenotype [34].

Recessive mutations in *BMP1,* which encodes metalloprotease BMP1 and its longer isoform mammalian tolloid, cause type XIII OI. BMP1 plus an enhancer protein affects the cleavage of the type I collagen C-propeptide. Type XIII OI is, however, more than the recessive enzymatic counterpart to the dominant structural mutations in the collagen cleavage site causing HBM OI. BMP1 is the C-propeptidase of types I–II–III procollagen and the N-propeptidase of type V collagen, as well as processing non-collagenous ECM components prolysyl oxidase, prodecorin, probiglycan and dentin matrix acidic phosphoprotein 1 (DMP1). Not surprisingly, recessive BMP1 mutations cause a more severe phenotype than HBM OI, ranging from mild to severe OI. Many of these patients have bone hypermineralization by qBEI. Incorporation of pC-collagen into ECM leads to abnormal organization of fibrils and may provide an increased number of sites for mineral nucleation [36–38].

All other genes in which mutations directly impact collagen post-translational modification, trafficking, polyadenylation etc. all fall into one of the following (mostly) recessive sets.

### Types V, VI, and Atypical VI OI- *IFITM5*, *SERPINF1*, *IFITM5*-p.S40L: Altered Bone Mineralization

In this grouping, dominant mutations in *IFITM5*/BRIL and recessive mutations in *SERPINF1*/PDEF both lead to OI with characteristic bone hypermineralization but without alterations in the structure or post-translational modification of type I collagen, although collagen secretion is decreased. Each set of mutations has distinctive histomorphometry focused on lamellar disorganization. A special *IFITM5* mutation with a phenotype similar in many respects to that caused by null mutations in PEDF, suggests that their mechanistic pathways are inter-connected and may converge with the biochemical and molecular cascade of events in OI initiated by collagen structural mutations.

A recurrent gain-of-function mutation in the 5’‐UTR of *IFITM5* (c.‐14C > T), encoding bone-restricted interferon-inducible transmembrane (IFITM5)-like protein (BRIL), is the cause of almost all instances of OI type V [39, 40]. This *IFITM5* mutation generates a new upstream start codon that results in addition of five amino acids (Met-Ala-Leu-Glu-Pro (MALEP)) to the N-terminus of BRIL (MALEP-BRIL) [39].

BRIL was discovered as a protein with increased expression during osteoblast differentiation, especially during the maturation/mineralization phase [41], using a signal trap screening system to detect new genes encoding proteins in bone. This function of BRIL was further corroborated by increased in vitro mineralization by type V osteoblasts derived from patient surgical bone chips [42], indicating a role of BRIL in bone mineralization. Further, MALEP-BRIL showed no differences in cell membrane localization, topology, and synthesis level compared to WT BRIL [43], supporting a gain-of-function mechanism.

Type V OI was defined radiographically and as the first type of OI that is not caused by mutations in collagen genes [44]. Type V OI is a dominantly inherited and moderately severe type of OI, which is highly variable in phenotype [45], characterized by hyperplastic callus formation predominantly in long bones after fractures or surgical interventions, ossification of the forearm interosseous membrane, radial head dislocation, and dense metaphyseal bands in the forearm. Blue sclerae and dentinogenesis imperfecta (DI) are absent. Polarized light microscopy revealed that type V OI Iliac crest bone has an irregular or meshlike appearance of lamellae [44]. Type V OI bone is hypermineralized, with BMDD (bone mineral density distribution) shifted towards increased mineralization, despite having no abnormality of collagen structure or modification [46]. In addition, type V OI bone has an increased osteocyte lacunar density, that, together with irregular bone lamellae, indicates altered bone remodeling [46]. There is currently no surviving murine model for type V OI. *Ifitm5*-KO mice showed a non-significant bone phenotype compared to patients with type V OI [47]. On the other hand, transgenic mice overexpressing *Ifitm5* in osteoblasts were perinatal lethal with skeletal defects, fractures, and delayed mineralization, in addition to impaired in vitro mineralization of osteoblasts [48]. Another mouse model for type V OI, with a knock-in mutation, was perinatal lethal as well, with hypomineralized skull, wavy ribs, shorter long bones and increased hypertrophic chondrocytes in midshaft of long bones. Calvarial osteoblasts from the knock-in mouse showed delayed differentiation and mineralization, and increased expression of inflammatory and adipogenic markers [49].

Type VI OI, an autosomal recessive disorder, is caused by null mutations in serpin family F member 1 (*SERPINF1*) gene, which encodes the secreted glycoprotein pigment epithelium–derived factor (PEDF) [50, 51]. Type VI OI is generally a severe progressive form of OI, with normal phenotype at birth, and presenting with long bone and vertebral compression fractures in early childhood. Affected individuals have white or faintly blue sclerae and absent DI. Type VI OI was also delineated before the causative gene was identified, based on bone histomorphometry with an irregular fish-scale pattern of lamellae visualized under polarized light, and excessive accumulation of osteoid reflecting increased mineral lag time [52]. This bone is also hypermineralized, although surrounded with areas of low bone mineral content and an increased number of osteocytes [53]. Patients with type VI OI have elevated alkaline phosphatase (ALPL) levels in serum as children [52]. In addition, circulating PEDF is undetectable in their serum [54]. PEDF is a potent endogenous inhibitor of angiogenesis [55], which binds to collagen type I in extracellular matrix. Binding to collagen is critical to activate the anti-angiogenesis function of PEDF; mutation of either the collagen-binding site on PEDF or the PEDF-binding site on collagen abolishes PEDF anti-angiogenic activity [56, 57]. Using a *Serpinf1*^*−/−*^ murine model [58], PEDF was shown to have a role in stimulation of osteogenesis and impairing bone vascularization, with TGF-β having the opposite trends [59]. This suggests that PEDF and TGF-β antagonism controls these two processes and contributes to type VI OI pathogenesis. The mechanism of type VI OI remains open for further investigation—competing models focus on the effect of anti-angiogenesis on cartilage ossification [60] or on the collagen-binding properties of PEDF, or even on the capacity of PEDF to induce osteoblast differentiation in cooperation with Wnt3a [61], proper transition to osteocytes and mineralized nodule formation [53].

Ten patients with heterozygous *IFITM5*, c.119C > T (p.Ser40Leu) mutation have been described with severe progressive OI that is not associated with radiographic or histologic findings of type V OI. These patients develop extremely short stature, vertebral compression fractures, scoliosis, bowed limbs [62–71]. Intriguingly, their clinical presentation is similar to type VI OI, including elevated unmineralized osteoid and a fish‐scale pattern of lamellation in bone tissue. Serum ALPL levels were elevated in childhood, although the patients do not carry PEDF mutations and the serum PEDF levels are normal. This surprising clinical outcome for an *IFITM5* mutation was named atypical type VI OI (aVI OI), connecting types V and VI OI and suggesting synergistic roles of BRIL and PEDF [62] since no evidence has been uncovered for a direct interaction between BRIL and PEDF. While WT BRIL and MALEP-BRIL were shown to attach to the plasma membrane via palmitoylated cysteine residues (C50 and C51), BRIL p.S42L was trapped in the Golgi and poorly palmitoylated, perhaps due to steric hindrance from the proximity of the palmitoylation sites [43]. The newly generated *Ifitm5*/BRIL p.S42L mouse, a knock-in mouse model for atypical type VI OI, has multiple long bone fractures, and decreased whole body BMD. Mechanical testing demonstrated increased bone brittleness [72]. Additionally, mice showed alterations of bone material properties including hypermineralization of the matrix, increased vascular porosity, elevated osteocyte lacunae density and disordered collagen fibril orientation by second-harmonic generation (SHG) [73].

### Types VII, VIII and IX OI-*CRTAP*, *P3H1*, *PPIB*—Procollagen prolyl 3-Hydroxylation Complex

Collagen has a triple-helical tertiary structure and undergoes post-translational modification of proline and lysine residues by prolyl 4-hydroxylase (P4H1) and LH1, an important step for proper folding and stability of the collagen helix [74]. Although the 3-hydroxylation of the α1(I)Pro986 residue had been known for several decades, neither its role nor the identity of the prolyl 3-hydroxylase was known. Isolation of prolyl 3-hydroxylase revealed that an ER-resident complex of 3 proteins was responsible for prolyl 3-hydroxylation of α1(I)Pro986 on each α1(I) chain of collagen types I and II [75, 76], specifically prolyl 3-hydroxylase 1 (P3H1), which accomplishes the enzymatic function, cartilage‑associated protein (CRTAP), the helper protein providing mutual stabilization to P3H1, and cyclophilin B (CyPB), the major collagen PPIase which here functions as a chaperone. The identification of the genes encoding the components of the prolyl 3-hydroxylation complex as the causative genes for 3 severe types of recessive OI demonstrated that the complex and its relatively minor modification have a major role in bone development [77].

The first evidence of an important role of CRTAP in bone was discovered by studies on *Crtap*-null mice, which developed osteochondrodysplasia with severe osteopenia and decreased osteoid formation [78]. Null and point mutations in *CRTAP* are responsible for development of type VII OI [78–82], a recessive form with a generally lethal outcome, although there are some very severe surviving patients. The distinctive clinical characteristic of type VII is a rhizomelia, reflecting the role of CRTAP in both cartilage and bone. Other features include fractures at birth, bowing of lower extremities, white or light blue sclerae and osteopenia.

Recessive type VIII OI is caused by null mutations in the *P3H1* gene that encodes P3H1, which was independently isolated as an extracellular matrix proteoglycan named leprecan [81, 83–85]. As for CRTAP, most individuals with type VIII OI have a perinatal lethal phenotype; severe survivors are more common for Type VIII than for type VII OI and share their rhizomelia. Recently, unusual dental anomalies such as hypodontia, a mesiodens, and single rooted second permanent molars were reported in type VIII OI [86]. Using *Crtap*^*−/−*^ murine model, it was shown that lung fibroblasts synthesize type I collagen with altered post-translational modifications, negatively impacting lung function [87].

In vitro studies of fibroblasts from types VII and VIII OI patients showed mutual stabilization of CRTAP and P3H1 in the ER collagen prolyl 3-hydroxylation complex. The studies revealed that both CRTAP and P3H1 proteins were absent or reduced in cells with null mutations in either gene, while rescue studies transfecting fibroblasts with *CRTAP* or *P3H1* constructs restored both CRTAP and P3H1 protein levels [88]. In addition, knock-in mice with an α1(I)P986A substitution that cannot be 3-hydroxylated revealed the role of the 3Hyp substrate site itself in recessive OI as affecting collagen cross-linking and structural organization. Mutant mice had otherwise normal bone phenotype; however, the bone collagen hydroxylysyl pyridinoline (HP) and lysyl pyridinoline (LP) crosslink ratio was doubled, with decreased collagen fibril diameter [89]. Complementary studies of knock-in mice with a single amino acid substitution in the active site of P3h1 showed P3h1 activity was suppressed but it was still able to form a complex with Crtap thus retaining the collagen chaperone function. This mutation also resulted in the absence of prolyl 3-hydroxylation at Pro986 of the α1(I) and α1(II) collagen chains [90].

CyPB, encoded by peptidylprolyl isomerase B (*PPIB*), is a peptidyl-prolyl cis–trans isomerase which is an ER resident [91]. It is multifunctional, with roles in inflammation, cancer and viral infection [92], as well as in procollagen folding and secretion [93]. Mutations in *PPIB* cause recessive type IX OI, with moderate to lethal phenotype, similar to *CRTAP* and *P3H1* mutations [94–96]. The role of CyPB in the 3-hydroxylation complex was illuminated by studies of a mouse model lacking *PPIB* expression [97]. *Ppib* knockout mice had decreased bone volume, bone mechanical properties and increased bone fragility. CypB-deficient osteoblasts had normal CRTAP levels, while levels of P3H1 were reduced by half, resulting in a marked reduction in collagen prolyl 3-hydroxylation and in site-specific changes in collagen lysyl hydroxylation and glycosylation. Intracellular folding of type I collagen in CyPB-null cells was slower than WT and collagen secretion was reduced; interestingly, collagen folding could be reduced further by the addition of cyclosporin A (CsA), suggesting some redundancy exists for the PPIase function required for collagen folding. The reduced lysyl hydroxylation of CypB-null cells is associated with a marked decrease of the HP/LP crosslink ratio, which is the opposite of the crosslink ratio alteration seen in classical OI caused by a collagen structural change [97]. The reduced prolyl 3-hydroxylation, changes in lysyl modification and altered cross-links may alter matrix assembly and ultimately bone stability and strength.

### Type X OI-*HSP47*, Type XI OI-*FKBP10*, and Type XXI OI—*KDELR2*-Collagen Folding and Cross-linking

Type X is an extremely rare type of recessive OI, with a severe to lethal phenotype. At birth, patients have severe skeletal defects, including thin ribs with multiple fractures, generalized severe undermineralization, platyspondyly, deformed long bones, hydranencephaly, macrocephaly, blue sclerae, and Wormian bones. Most presented with sudden early death due to respiratory issues [98–100]. Because of the rarity of Type X OI, information on the histomorphometry and mineralization of bone tissue has not been reported.

Type X OI is caused by homozygous mutations in serpin family H member 1 (*SERPINH1*) gene, which encodes heat shock protein 47 (HSP47). HSP47 is a collagen- specific chaperone which resides in the ER and facilitates folding of collagen chains [101]. It binds to fully folded triple helical procollagen in the ER, then dissociates in the *cis*-Golgi, preventing procollagen aggregation [102]. *Hsp47*-knockout mice are embryonically lethal [103], with reduced production of mature collagen types I and IV, and accumulation of type I collagen in the ER of *Hsp47*^−/−^ cells [104]. Collagen secreted from cells with HSP47 defects has increased protease susceptibility, suggesting it is not properly folded [98]. In a recent study, a type X OI child with a homozygous mutation in the collagen interacting surface of HSP47, which reduced its affinity to type I collagen, was shown to have increased post-translational modification of type I procollagen, with significant upregulation of several chaperones (FKBP65, PDI, P4HA1, CypB, P3H1, CRTAP, P3H3, SC65, LH1 and LH3) involved in modification and folding of procollagen as part of a larger compensatory mechanism [100].

Another molecular chaperone, FKBP65, which is a PPIase, also resides in the ER and interacts with collagen [105]. Mutations in the gene that encodes FKBP65, *FKBP10,* cause either recessive type XI OI, Bruck syndrome or Kuskokwim syndrome. The initial report of homozygous mutations in *FKBP10* presented patients with moderately severe OI with progressive kyphoscoliosis, long bones fractures with severe deformities, ligamentous laxity, grayish sclerae and no DI [106]. FKBP65 was shown to have a role in collagen cross-linking since patient cells deficient in *FKBP10* had normal folding of type I collagen, but significant reduction of collagen cross-linking and collagen deposition in the matrix [107, 108]. On other hand, mutations in *FKBP10* were shown to be a cause of Bruck syndrome [109–111], a rare recessive disorder similar to OI, characterized with congenital joint contractures, bone fragility, osteoporosis and short stature. Recent study showed that novel variants in *FKBP10* caused Bruck syndrome with orodental changes such as dental malocclusion and enamel hypoplasia [112]. Finally, Kuskokwim syndrome, in which affected individuals have congenital contractures with minimal skeletal manifestations, is caused by an in-frame deletion of a conserved tyrosine in the third PPIase domain of FKBP65 [113].

There is an interesting relationship between the proteins responsible for types X and XI OI. Cells with HSP47 mutations have decreased FKBP65, and procollagen aggregates in ER-adjacent compartments, possibly autophagosomes [114]. While absence of FKBP65 does not affect HSP47 levels, it does result in mis-localization of HSP47 [114]. Apparently, the two chaperones cooperate in procollagen trafficking from the ER to the Golgi.

Type XXI OI, caused by recessive defects in KDELR2, intersects with type X OI, caused by deficiency of HSP47. Patients with type XXI OI have a severe progressive deforming OI [115, 116]. Fewer than ten case, ranging from a preterm fetus to the 5th decade of life have been reported, with multiple fractures, long bone and chest deformities, wheelchair dependence, and short stature. Blue sclerae, dentinogenesis imperfecta and neurodevelopmental delay are present in some individuals.

These individuals have homozygous missense or frameshift mutations in KDELR2, which encodes the KDEL receptor 2. This receptor, a member of the KDEL receptor family, is localized in the cis-Golgi, the ER, and the intermediate ER-Golgi compartment (ERGIC). The receptors are part of the COPI vesicles involved in retrograde transport of ER-resident proteins containing the signal peptide Lys-Glu-Asp-Leu (KDEL) from the cis-Golgi back to the ER in a pH-dependent process [117, 118]. KDELR1 and 3 cannot compensate for loss of KDELR2. Thus, loss-of-function defects in KDELR2 result in secretion of the normally ER-localized type I collagen-specific chaperone HSP47 to the extracellular space. When extracellular HSP47 interacts with collagen molecules in matrix, it binds to monomeric and multimeric collagen and impairs their fibrillogenesis, yielding thin fibrils with excess bound HSP47, but not does alter collagen modification [116]. Interestingly, XXI OI fibroblasts have reduction of both intracellular and secreted type I collagen. Depleted intracellular HSP47 also results in reduction of intracellular FKBP65, overlapping type XXI OI with type XI OI, as well as type X [116]. The *Kdelr2*^*−/−*^ murine model has premature lethality; its bone structure appears to model the patient skeletal phenotype, as well has having cleft palate [119].

The remaining sections are about OI types that have an indirect collagen-related mechanism through changes in osteoblast development.

### Type XII OI-*SP7*—Osteoblast Differentiation and UPR Pathway

Homozygous or heterozygous mutations in *SP7* are the cause of extremely rare type XII OI, with either with recessive [120–123] or dominant inheritance [124] and characterized by predominantly moderate bone fragility and deformity, delayed eruption of teeth, normal sclerae and variable dentinogenesis imperfecta. The first reported individual had a homozygous frameshift mutation, moderately severe OI and died early in childhood of respiratory etiology [120]. The same homozygous mutation was reported later in a 17 year old Chinese boy [125]. Recently, a homozygous p.Arg316Cys substitution, which alters the first zinc finger domain in *SP7,* was identified in independent reports [121, 126]. First, a 13-year old Iraqi boy had short stature, bone fragility fractures, scoliosis, hearing loss and craniofacial anomalies. A bone biopsy showed increased cortical porosity and increased trabecular bone turnover. Second, Kuwaiti male siblings in their early 20 s have moderately severe OI, stature in the normal range, several long bone fractures, dentinogenesis imperfecta, conductive hearing loss and craniofacial anomalies [126].

Intriguingly, there have also been reports of pathological heterozygous mutations in *SP7*. Siblings with a heterozygous substitution in a highly conserved zinc finger domain of osterix (p.Glu340Ala) share low cortical density with the recessive OI, but have low bone turnover [124] rather than the high bone turnover seen in *SP7* recessive OI. Unrelated patients were reported with different phenotypic outcomes of a heterozygous p.Ser309Trp substitution, one patient with cranial hyperostosis, craniosynostosis, long bone fragility, and high bone turnover [127] and one with Juvenile Paget’s Disease [128].

Further investigations of the cellular and biochemical features will be required to provide understanding of the variable outcome of inheritance and phenotype of Type XII OI.

*SP7* gene encodes Osterix, a zinc finger transcriptional factor important for osteoblast differentiation and maturation. Osterix interacts with Runx2 to induce *COL1A1* gene expression [129]. It also induces expression of antagonists of the Wnt pathway, sclerostin and DKK1 [130]. Conversely, activation of this pathway may also play an important role in classical OI with ER stress, since the IRE1a-XBP1 branch of the UPR promotes *SP7* transcription [131].

Mice with an *SP7* deletion die shortly after birth with absent bone development due to lack of osteoblast differentiation [132].

### Type XIV OI-*TMEM38B*-Collagen Production and Modification, Cell Energetics and Attachment

Type XIV OI, with recessive inheritance, is caused by mutations in the *TMEM38B* gene, encoding the ER membrane trimeric intracellular cation channel type B (TRIC-B) [133–136]. These are predominantly null mutations caused by deletions; other mutations include splice site variant mutations in *TMEM38B* [137, 138]. Type XIV OI is characterized by a broad phenotypic range, from asymptomatic to severe. The common clinical presentation consists of bone fragility, frequent fractures, bowed limbs and severe osteopenia. Type XIV OI bone is distinctive in remodeling compared to other OI types. Typically, OI bones are hypermineralized and exhibit high bone turnover. However, type XIV OI has low bone turnover with low osteoblast and osteoclast numbers, with mainly normal bone matrix mineralization [139].

TRIC-B protein, a monovalent cation channel, together with inositol 1,4,5-trisphosphate receptor (IP3R), is important for regulating calcium flux from the ER to cytoplasm [140]. Ca^2+^ signaling is essential for collagen metabolism. *TMEM38B*-null osteoblasts and fibroblasts have been shown to have impaired ER-Ca^2+^ flux kinetics, which induces ER stress, thus affecting collagen synthesis and secretion. In addition, although LH1 was increased and calcium-dependent FKBP65 was decreased in TRIC-B cells, absence of the TRIC-B channel caused reduction in hydroxylation of lysines in the collagen helical region and increased telopeptide hydroxylation. Procollagen chain assembly was delayed, causing retention of misfolded collagen in *TMEM38B* deficient cells and ultimately affecting the collagen assembly in matrix [136]. Recently, it was shown that deletion of *TMEM38B* in osteoblasts impacts a broader range of calcium-dependent cellular processes, emphasizing a novel role of TRIC-B in osteoblast differentiation and mineralization. The prolonged ER stress resulting from the absence of TRIC-B decreases the expression of mitochondrial fusion/fission markers, resulting in strikingly elongated mitochondria. Furthermore, increased generation of reactive oxygen species (ROS) by mitochondria demonstrates their functional impairment. Contact sites between ER and mitochondria (ER-MCSs) regulate mitochondrial fusion/fission, calcium, and lipid trafficking, important processes for the maintenance of mitochondrial physiology, further emphasizing the indirect but important role of TRIC-B in mitochondria function. This was the first evidence of mitochondria changes in osteoblasts from OI types with recessive inheritance. In addition, deletion of TRIC-B disrupted cell adhesion of osteoblasts, further affecting the cell–cell communication such as gap and tight junctions, cell proliferation and cell cycle [141].

*Tric-b* knockout mice die shortly after birth due to respiratory distress [142], and have decreased bone mineralization and altered collagen deposition [143]. Recent murine studies in a new *Tmem38b* knockout mouse model demonstrated that absence of the TRIC-B channel affected skeleton growth, as well as osteoblast differentiation and collagen synthesis, due to reduced calcium calmodulin kinase II-mediated and TGF-β signaling [144].

### Type XV OI-*WNT1*-Osteocyte-Specific Function

The Wnt/β-catenin pathway is a major pathway regulating the bone development. Wnt 1 is a secreted glycoprotein; palmitoylation of WNT1 at Serine224 is required for intracellular trafficking and full protein activity [145]. Mutations in *WNT1* can cause either recessive or dominant type XV OI [146–158]. Homozygous mutations in *WNT1*, causing recessive OI, are characterized with fractures, short stature, osteoporosis, vertebral compression, and variable neurological problems, while heterozygous mutations in *WNT1*, causing dominant OI, present with early-onset osteoporosis. In vitro studies showed that Wnt1 protein with either homozygous or heterozygous mutations could not activate LRP5-mediated WNT-regulated β-catenin signaling [147, 149, 158].

*Wnt1*-null mice die postnatally due to diminished development of central nervous system [159]. The swaying mouse (*Wnt1*^*sw/sw*^), which carries a spontaneous single nucleotide deletion in *Wnt1*, was shown to have similar skeletal characteristics as patients with type XV OI [160]. The mice exhibited fractures, osteopenia, reduced bone strength and decreased osteoblast function. Wnt1 expression in bone is surprisingly low, but expression during neurological development is high [159]. The studies on the late-osteoblast-specific and osteocyte-specific WNT1 loss- and gain-of-function mouse models further illuminated the role of osteocyte-specific WNT1 in bone. *Wnt1* absence in osteocytes induced low bone mass with fractures, while *Wnt1* overexpression increased bone formation, partially mediated through mammalian target of rapamycin complex 1 (mTORC1) signaling [161].

### Type XVI OI-*CREB3L1*, Type XVIII OI-*MBTPS2*—the RIP Pathway Affects Bone

Type XVI and XVIII OI revealed an unexpected role for Regulated Intramembrane Proteolysis (RIP) in bone development. RIP had previously been extensively studied, including the seminal work of Brown and Goldstein, for its role in cholesterol metabolism. Now defects in Oasis, a substrate for RIP processing, and in S2P, the second of two sequentially acting Golgi membrane-embedded proteases which activate a variety of transcription factors by cleaving them, have pointed to a significant role in bone.

Recessive type XVI OI is caused by mutations in cAMP responsive element binding protein 3-like 1 (*CREB3L1*) gene, which encodes OASIS (old astrocyte specifically induced-substance). The phenotype of type XVI shows a substantial variability from mild to perinatal lethal [162–168]. The initial report of type XVI OI involved a Turkish family with two affected children, one with in utero fractures and bowed limbs who died at 9 month and other terminated in utero with thin ribs and fractures revealed by autopsy [162]. *Oasis*-null mice were shown to develop severe osteopenia and fractures, with reduced type I collagen in bone and osteoblasts, but not in dermal fibroblasts. OASIS is a transmembrane basic leucine zipper (bZIP) transcriptional factor, residing in the ER, which under stress conditions relocates to Golgi membrane and undergoes cleavage by RIP, releasing its N-terminal domain which induces gene transcription in the nucleus (169). OASIS binds to the *Col1a1* gene promoter via unfolded protein response element (UPRE)-like sequence and increases its transcription [170]. OASIS also regulates transcription of SEC24D, which is involved in ER-Golgi trafficking [166].

Type XVIII OI, the first X-linked recessive OI, is caused by missense mutations in membrane-bound transcription factor site-2 protease (*MBTPS2*) gene, encoding S2P. In OMIM (Online Mendelian Inheritance in Man), gene defects in *MBTPS2* are listed as type XIX OI (#301014). S2P catalyzes the second of two sequential cleavage reactions in the RIP process, following S1P cleavage, that occur in the Golgi membrane when ER-stress and other lipid-related processes cause the transport of transcription factors from the ER membrane to the Golgi.

Type XVIII OI is characterized by short stature, bone fractures and bowing, barrel chest and vertebral compressions and scoliosis [171]. It is noteworthy that the published and unpublished S2P mutations causing OI are all localized to residues in the NPDG motif that is crucial for zinc ion coordination. Dozens of other missense mutations in S2P occur throughout the remainder of the protein, but especially in the active site, and have been shown to induce cholesterol-related conditions—ichthyosis follicularis, atrichia, and photophobia (IFAP), BRESEK/BRESHECK syndrome, and keratosis follicularis spinulosa decalvans (KFSD) [172–174]. It is interesting that recent omics studies of S2P patient skin fibroblasts [175] or fibroblasts from a proband umbilical cord [176] showed upregulation of fatty acid pathways [175]. However, OI Type XVIII patients do not have evidence of cholesterol-related conditions, nor do IFAP/KFSD patients have symptoms of OI. Given the prior demonstration that transcription factors related to lipid, such as SREBPs, are also not well-cleaved in type XVIII OI-affected osteoblasts [171], this may speak to a tissue-specific critical function distal to RIP cleavage of OASIS that makes a specific set of S2P mutations crucial for bone formation. In type XVIII OI, not only is collagen expression reduced, but also hydroxylation of the α1(I) and α 2(I) Lys87 residue that is crucial for collagen cross-linking is reduced by half, weakening bone strength [171].

Furthermore, mutations in S1P, which cause a full absence of S1P/S2P cleavage, have also been reported to affect the skeletal system. A patient reported with a recessive mutation in the *MBTPS1* gene, encoding S1P, has features of a skeletal dysplasia, including growth retardation, kyphoscoliosis, and dysmorphic facial features, but intriguingly not OI [177]. Cartilage-specific S1P knockout mice, which die shortly after birth, were shown to develop chondrodysplasia with absent endochondral ossification [178]. Further investigation of these very rare patients and important murine models are needed to understand the full complexity of the RIP system, its substrates and their interactions.

### Type XVII OI-*SPARC* Matrix Protein with Intracellular and Matrix Functions

Homozygous mutations in secreted protein acidic and rich in cysteine (*SPARC*) are responsible for causing the extremely rare recessive type XVII OI. Patients with type XVII OI usually present normally at birth, developing severe progressive OI in early childhood, with frequent long bones fractures, vertebral compression fractures, muscular hypotonia, and scoliosis [122, 179, 180]. Their bone tissue is hypermineralized, as in classical OI [179]. SPARC protein or osteonectin was identified over 40 years ago as a bone-specific protein important for mineralization [181]. It is a secreted matricellular glycoprotein that binds different ECM proteins including collagen in a calcium-dependent manner [182]. It is interesting that defects in a protein component of matrix have recessive inheritance, which points to its pathology being based on disruption of interactive rather than structural functions. The residues critical for type XVII OI are located in the EC domain of osteonectin and form a salt bridge critical for Ca^2+^-binding and subsequent collagen binding. Osteonectin has also been proposed to have other intracellular and matrix functions. Cells from type XVII OI patients secrete reduced amounts of collagen, which is partially overmodified, supporting a collagen chaperone function for osteonectin assisting HSP47 [179]. SPARC-null mice exhibited osteopenia and decreased bone formation and bone remodeling [183]. In addition, SPARC was shown to affect murine collagen fibril formation in skin, producing smaller fibrils [184], perhaps via increased activity of transglutaminase for cross-linking of matrix proteins [185].

### Type XIX OI—*TENT5A*/FAM46A Post-Transcriptional Regulation of Collagen Production

A small number of patients with a severe to lethal skeletal dysplasia have been reported to have homozygous or bialleleic missense or frameshift mutations in *TENT5A*, and designated autosomal recessive type XIX OI [186]. In OMIM, gene defects in *TENT5A* are listed as type XVIII OI (# 617952). *TENT5A* encodes FAM46A, a member of the nucleotidyltransferase (NTase)-fold protein family of non-canonical poly(a) polymerases, which are conserved in all known animal genomes [187].

This newer addition to the genetic etiologies of OI fulfills its expected collagen-related function by its involvement in the polyadenylation of transcripts for *Col1a1* and *Col1a2* in murine studies [188]. However, the collagen in type XIX OI bone matrix is disordered, as well as decreased, suggesting significant indirect contributions to the bone defect. FAM46A mutations causing type XIX OI are located in the conserved DUF1693 domain and include both missense substitutions and stop codons [186]. Distinction between misfunction and loss-of-function cannot yet be made since neither transcript levels nor western blots from patient cells have been reported. In addition, studies of Type XIX OI bone tissue by histomorphometry and BMDD will be critical in the future.

The diagnosis of type XIX OI is made in the first months to years of life, based on typical features such as congenital bowing of limbs, early fractures which accelerate in frequency in childhood, blue sclerae, hyperlaxity and motor delay [186, 189]. Some have short stature and dental abnormalities. Vertebral compressions and Wormian bones are present on radiographs.

Because the children with type XIX OI were first detected by screening a group of patients with Stuve-Wiedmann Syndrome [190], with whom they share congenital bowing and progressive scoliosis, the assignment of this gene as OI-causative was cautiously undertaken. Now, the combination of patient data, murine phenotype and molecular data on polyadenylation of type I collagen transcripts has made the assignment to OI nosology clear.

The *Fam46a* null mouse, which carries a homozygous null mutation, was generated by ENU mutagenesis before *TENT5A* was proposed as an OI-causative gene [191]. Its small size, long bone abnormalities, with bent and twisted limbs, compressed rib cage, and thin cranium support the skeletal dysplasia assignment of the gene defect. Long bone fragility, as well as thin cortices and reduced trabecular bone were reported. In mouse, *Tent5a* is expressed in both newborn and adult bone tissues, suggesting both prenatal roles in bone development and post-natal roles in bone remodeling. Murine calvarial osteoblasts have impaired mineralization in vitro. The murine osteoblast collagen secretion deficiency is reduced to the level of a collagen null allele. One might have expected a rather mild skeletal dysplasia from a straightforward quantitative reduction of normally modified type I collagen. However, the collagen in murine bone tissue is strikingly abnormal, with thinner and disorganized fibrils. This may be related to the broader role of FAM46A, which polyadenylates a set of cytoplasmic mRNAs encoding proteins impacting bone development and which are secreted coordinately with bone mineralization [188]. Fifty-two mRNAs in *Tent5a* KO osteoblasts had significantly shortened poly-A tails; the transcripts at the top of the list were *Col1a1*, *Col1a2*, *SerpinF1* and *Sparc* [188]. Yet another function of FAM46A which may contribute to the bone dysplasia of type XIX OI is its role as a binding partner for SMADs in the TGF-β pathway, physically interacting with Smad1 and Smad4 to stabilize them, and thus promoting BMP target expression [192]. The relative contribution of significantly reduced collagen, reduction of other bone matrix proteins which are related to OI types, and presumptive reduction in BMP signaling has not been investigated.

In addition, the association of FAM46A defects with other conditions remains intriguing. FAM46A was first described as a candidate gene for human retinal disease; expression of *TENT5A* in retina underlies the recessive retinitis pigmentosa associated with novel VNTRs (variable number tandem repeats) in exon 2 [193]. Elsewhere in the body, *TENT5A* exon 2 VNTRs are associated with skeletal conditions such as osteoarthritis of hip and knee [194] and adolescent idiopathic scoliosis [195], an assignment made reasonable by the strong expression of *TENT5A* in human and murine osteoblasts.

### Type XX OI—*MESD* Disrupting the Intersection of Collagen and LRP5/6 and Wnt Signaling

Type XX OI, a severe to lethal recessive skeletal dysplasia, is caused by biallelic or frameshift mutations in exons 2 or 3 of *MESD* [196–198]. Some affected individuals have been stillborn. Common features reported in more than a dozen children who survived have included an initial fracture by age 2 years, with a progressive deforming course. When young, they have a low birth weight and blue sclerae, and radiographic evidence of Wormian bones, gracile ribs and severe overtubulation. By the second decade of life, dental abnormalities, such as disorganized dentition and oligodontia [196, 197], are prominent. They have short stature and rhizomelia or micromelia is prevalent. Progressive scoliosis and kyphosis are associated with vertebral compressions. Type XX OI patients often have intellectual disability [196, 199], a relative rarity among OI types, previously reported in some individuals with type XV OI, due to *WNT1* defects, as well as type XXI OI with *KDELR2* mutations, in which neurodevelopmental disorders were reported [115].

The *MESD* gene encodes MESD (mesoderm development candidate 2), which functions as a chaperone of the low-density lipoproteins in the ER [200]. The critical function of MESD for the correct localization of LRP5/6 at the cell surface puts type XX OI at the overlap of pathways leading to OI or to LRP5/6-related conditions. Generally, the mutant MESD protein cannot be retained in the ER, although it is expressed and has similar stability and chaperone function as normal MESD [196, 201]. The mis-localization of LRPs results in their aggregation in the cytoplasm [200, 201], causing loss of normal signaling. For example, the oligodontia that is distinctive to this OI type has been speculated to arise from reduced LRP6 signaling.

While the consequences of overlap of type XX OI and LRP5/6 conditions can be dissected, it is clear that bone with MESD defects is distinctive from most OI types [198]. One of the salient features common to most OI dominant and recessive types, the hypermineralization of bone when assayed by BMDD, is not present. Instead, BMDD of MESD bone matrix shows lower and less homogeneous mineralization than control bone but has islands of hypermineralized cartilage remnants. There are an increased number of irregularly shaped lacunae, connected by broad canaliculi. Though mindful of the distinctiveness of type XX OI, it also fulfills the OI biochemical expectation of being type 1 collagen-related. MESD has recently been shown to be a direct chaperone of pro-α1(I) [201]; consequently, cells with mutant MESD have cytosolic aggregates of type I collagen that block intercellular nanotubes, resulting in significant procollagen intracellular retention. To degrade the collagen aggregates, patient cells increase autophagy, as confirmed by co-localization of aggregates with LC3, an autophagy marker, and impaired collagen degradation in cells treated with bafilomycin A [201]. Failure to fully compensate for the retained aggregates leads to an overall cellular stress. This is notably different from the ER stress response that occurs in OI patient cells with collagen misfolding or retention of overmodified collagen.

It is also interesting that the non-secreted aggregates of type I collagen are mechanistically related to the type XX OI phenotypic feature of dermal aging-related skin laxity. Low co-localization of pro-α1(I) with integrin subunit β1 (ITGB1) and deficiency of paxillin-positive foci in patient but not control cells underlie the weak attachment of proband fibroblasts with matrix surface [201]. It is notable that impaired cell–cell and cell–matrix attachment were also recently described in osteoblasts with type XIV OI, caused by impaired intracellular calcium flux in the absence of the *TMEM38B* channel [141].

Finally, a connection can be made back to WNT signaling, consistent with the intellectual and neurological aspects of type XX OI. Since MESD is a chaperone for LRP5 and LRP6, their aggregation in patient cells may disrupt WNT signaling. In fact, expression of BMP2 and BMP4 were strikingly downregulated, connecting types XX and XV OI [201]. Thus, the failure of mutant MESD to be retained in the ER leads to its inadequate availability as a chaperone for type 1 procollagen, LRP5, and LRP6, all of which aggregate in the cytoplasm, leading to cell stress, impaired matrix attachment and disruption of WNT signaling.

### Type XXII OI—*CCDC134*, OI Overlap with MAPK/ERK Signaling

Homozygous missense mutations in the first codon of *CCDC134* cause a severe recessive skeletal fragility syndrome that has been given the designation OI type XXII [202–204]. These defects, which have been reported in only a handful of patients, share pre- and post-natal short stature, multiple fractures and bowing of long bones, low bone density and Wormian bones. Three patients had pseudoarthroses while scleral hue has been variable. Patient bone histomorphometry reveals the decreased BV/TV typically found in OI, associated with atypical increased cortices, with normal mineral apposition rate (MAR) and bone formation rate (BFR), more characteristic of increased extracellular signal-regulated kinase (ERK) signaling [202].

The missense mutation responsible for type XXII OI alters the start codon of *CCDC134,* resulting in a loss of function of this secreted protein. CCDC134 contains a coiled-coil domain and is involved in some mitogen-activated protein kinase (MAPK) signaling pathways, as well as being responsible for the inhibition of phosphorylation of ERK and C-Jun N terminal kinase (JNK) [205]. Thus, primary osteoblasts derived from individuals with type XXII OI have increased ERK1/2 phosphorylation, reduced expression of osteopontin and *COL1A1*, and reduced mineralization in vitro [202]. This makes type XXII OI another OI form which contains elements of both OI and another set of skeletal dysplasias, in this case, bone dysplasias with dysregulation of the MAPK/ERK pathways [206], including Noonan syndrome, neurofibromatosis type 1, and cardiofaciocutaneous syndrome. ERK1/2 activation and decreased osteoblast differentiation have also been demonstrated in the classic “dripping candle wax” form of melorheostosis, although melorheostosis is caused by *MAP2K1* somatic mosaicism [207]. Given that ERK activation is important for osteoblast differentiation and maturation, the situation in type XXII OI appears more complex, with reduced osteoblast collagen and mineralization. Investigation of these pathways in OI XXII will be important to clarify how *CCDC134* mutations result in different outcomes, perhaps with overlapping pathways. *Ccdc134*^*−/−*^ mice have not been informative, since they die at E12.5–E14.5 of cerebral hemorrhages and their bone properties cannot be studied [208]. For the present, diagnostically grouping these mutations with recessive OI is the most appropriate course.

### Intracellular and Matrix Consequences of Different OI Mutations

The trafficking of collagen molecules is a complex process, consisting of anterograde and retrograde transport, which is dependent on the proper functioning of chaperone molecules to facilitate the collagen transport. After post-translational modifications and folding of the triple helix are completed, procollagen is transported anterograde from the ER to the Golgi through COPII vesicles [209]. For the transport of large molecules such as collagen, COPII vesicles require the transmembrane protein transport and Golgi organization 1 (TANGO1); null mutations of *MIA3*/TANGO result in procollagen retention in the ER [210]. Conversely, retrograde transport returns certain proteins back to the ER to fulfill their function. KDEL receptors, among others, function to mediate the sorting of ER proteins by binding COPI vesicles [209]. Studies of KDELR2 mutations, which cause severe progressive deforming type XXI OI, highlight its crucial role in collagen transport [116].

Misfolded collagen chains, often with increased post-translational modification, are either secreted slowly from cells or retained in the ER, causing ER stress and activation of UPR. UPR facilitates structure restoration of retained proteins. However, if the repair is not possible, the misfolded proteins are degraded either through ER-associated degradation (ERAD), which consists of ubiquitination and degradation by the proteasome, or through autophagy, a lysosomal self-digestion. The induction of ER stress and the subsequent activation of UPR pathways were reported in several OI types, both dominant and recessive. Some but not all mutations in *COL1A1* and *COL1A2* caused ER accumulation of collagen, increased expression of binding immunoglobulin protein (BiP), a chaperone that directly binds to collagen to support its folding and assembly, as well as UPR activation, autophagy and apoptosis in OI fibroblasts [211]. The same findings were reported in OI patient fibroblasts with mutations in *CRTAP, P3H1* and *PPIB* impairing prolyl 3-hydroxylation [212]. Both studies showed that the application of chaperone 4-phenylbutyrate (4-PBA) decreased UPR and ameliorated the cellular homeostasis. Recessive type XIV OI, caused by null mutations in the *TMEM38B*/TRIC-B channel, causes significant collagen accumulation in both osteoblasts and fibroblasts in the ER, inducing ER stress and UPR with a significant increase of BiP [136]. In recessive OI types involving RIP, there is a distruption of the process by which transcription factors such as OASIS are translocated to the Golgi membrane upon ER stress, cleaved by S1P/S2P, and transported to the nucleus for transcription of genes for ER-stress response or UPR pathways [169]. Thus, to which extent ER stress and UPR contribute to the OI phenotype, versus helping to mitigate it, remains unresolved.

One of the most intriguing recent findings is mitochondrial involvement in OI pathophysiology. Mitochondrial dysfunction was first observed in muscle tissue of OI murine models such as *oim*/*oim,* with evident reduction of respiration rates, biogenesis markers, mitophagy and electron transport chain components [213]. In a transcriptomics analysis of muscle in Jrt and *oim*/*oim* mice, *Mss51*, a mitochondrial metabolic stress inducible factor, was shown to be downregulated [214]. Recent studies of osteoblasts from dominant *COL1A2* G610C OI mice, showed that integrated stress response (ISR), induced by ER collagen accumulation, was regulated by expression changes in mitochondrial Hspa9/HSP70 (mt-HSP70) [215]. Recently, it was found for the first time that mitochondrial dysfunction is a contributing factor to OI osteoblast pathology. Deletion of *TMEM38B*/TRIC-B in type XIV OI osteoblasts induced striking changes in mitochondrial morphology, which became very elongated, developed crystolysis and increased production of superoxide [141]. Expression of mitochondrial fission/fusion markers was decreased, consistent with mitochondrial morphology and with transmission of ER stress through the contact sites between ER and mitochondria (ER-MCSs). The contact sites regulate various mitochondrial functions, including mitochondrial fusion/fission, calcium and lipid trafficking [216]. Fission and fusion components form hotspots and colocalize at ER-MCSs [217], which are enriched with IP3Rs, implicating the role of contact sites in regulation of mitochondrial calcium levels. Investigation of the role of mitochondria in OI bone pathology is likely to yield further exciting findings.

The stability of collagen in matrix is dependent on intermolecular cross-links that are formed by hydroxylation of lysine residues, carried out by lysyl hydroxylases (LH1, LH2, and LH3), encoded by *PLOD1*, *PLOD2*, and *PLOD3*, respectively. Abnormal collagen cross-linking is reported in OI and Bruck syndrome (skeletal disorder similar to OI with congenital joint contractures) via mechanisms of underhydroxylation and overhydroxylation of collagen telopeptide lysines [218, 219].

The main cause of increased fragility of OI bone is abnormal bone material properties, especially hypermineralization of bone matrix. The increased mineralization of bone matrix occurs in most types of OI and is one of the unifying feature of OI bone. Quantitative backscattered electron imaging (qBEI) is used to directly determine the bone mineralization density distribution (BMDD). qBEI studies showed increased bone mineral content in patient with dominant [32, 46, 220, 221], as well as in recessive OI types [53, 222–224]. Only exception to this rule are types XIV (*TMEM38B*) and XV (*WNT1*), with mostly normal mineralization, and XX (*MESD*) in which patients exhibit reduced bone mineralization [139, 225, 226].

OI bone has been shown to contain a higher number of osteocytes [227] and the resulting increase in porosity also contributes to bone fragility. This finding was consistent with investigations of the *Crtap*^−/−^ recessive mouse model, which demonstrated increased TGF-β signaling [228]. Furthermore, the osteocyte transcription was found to be significantly dysregulated in the *Crtap*^−/−^ mouse, with major changes in both WNT and TGF-β signaling [229], suggesting an important role of osteocytes in OI pathology. See sections on these types below.

The major cause of mortality and morbidity of patients with OI is respiratory dysfunction [230]. It was thought that impaired pulmonary function was extrinsic to the impaired collagen, caused by presence of scoliosis in OI patients [231]. However, studies showed that lung disease and some cardiac issues develop independently of scoliosis [232]. Pulmonary function was shown to decline in children with classical OI who did not have scoliosis [22]. More recently, the lung tissue abnormalities in OI were shown to have an intrinsic etiology, which can be exacerbated by scoliosis [233]. In this study of children and young adults with classical OI, all patients with types III and half with type IV had lung restriction, and many had parenchymal defects such as atelectasis and reticulation. As a result of functional defects caused directly by collagen abnormalities, 90% of patients in the study group had reduced gas exchange. Further, decreased forced expiratory flow (FEF) 25–75% and significant thickening of small bronchi wall indicate a critical role for small airways, the mechanism of which is currently not known [233].

Aortic root dilatation and valvular dysfunction in OI patients has been extensively investigated [234–237]. Patients with OI have a higher risk of cardiovascular abnormalities, such as valvular heart disease, heart failure, and aortic root dilation [238]. A single study reported on atrial fibrillation and hypertension in patients with OI [239]. Further studies are needed to understand the underlying mechanism of cardiovascular abnormalities in OI patients.

## Conclusions

The genes responsible for OI have now largely been defined. While several rare types may emerge, they will not pertain to very many individuals and will most likely be related to pathways containing the known genes. The identification of genetic defects causing OI and their mechanism are important. Further research on mechanism of the types and their interrelatedness will hopefully lead to a more encompassing conceptualization of OI pathophysiology, and ultimately to broad therapies.

Nosology, on the other hand, is not the equivalent of scientific data. It is an organization of the information at hand at the time, and, since that information changes with time, so will nosology. A particular nosology will be utilized if it represents a convenient organization for understanding the *status quo* and moving research forward. While it is possible that different nosology might be more useful to researchers versus clinicians and families, we propose that a dual or combination nosology might well serve both communities and even facilitate cross-talk. For example, the use of a genetic type in combination with an OI severity label based on both skeletal and non-skeletal features, would keep the distinctions of genetics in the forefront and clarify phenotypic variability. The shared goal is the use of genetics to understand the cause and change the outcome.
